# Evaluation of SARS-CoV-2 vaccination in pregnant and breastfeeding women

**DOI:** 10.1016/j.ijregi.2023.06.002

**Published:** 2023-06-14

**Authors:** Harvanova Terezia, Kobliskova Zuzana, Novak Petr

**Affiliations:** a Faculty of Pharmacy, Comenius University, Department of Organisation and Management in Pharmacy, Bratislava, Slovakia; b Institute of Neuroimmunology, Slovak Academy of Sciences, Bratislava, Slovakia

**Keywords:** Covid-19, Vaccine, Pregnancy, Breastfeeding, Safety, mRNA

## Abstract

**Objectives:**

We aimed to assess the safety experience of women vaccinated with vaccines against SARS-CoV-2 during breastfeeding or pregnancy.

**Methods:**

Via an online questionnaire, we have inquired about the safety experience of 2192 pregnant or breastfeeding women vaccinated against SARS-CoV-2, including the incidence of adverse events (AEs), pregnancy outcomes, and confirmed infection with SARS-CoV-2.

**Results:**

The incidence of other AEs was higher in women vaccinated during breastfeeding. Significant differences were observed for fatigue (relative risk [RR] 1.230, 95% confidence interval [CI] 1.051-1.444, *P* = 0.0098), headache (RR 1.822, 95% CI 1.379-2.418, *P* <0.0001), myalgia (RR 1.633, 95% CI 1.269-2.110, *P* <0.0001), chills (RR 2.027, 95% CI, *P* <0.0001), subfebrile temperature ≤38°C (RR 1.697, 95% CI 1.240-2.335, *P* = 0.0007), arthralgia (RR 1.924, 95% CI 1.340-2.776, *P* = 0.0002), fever >38°C (RR 6.410, 95% CI 2.890-14.30, *P* <0.0001), and shivers (RR 2.204, 95% CI 1.264-3.863, *P* = 0.0049). No pattern of serious AEs emerged. Menstrual cycle bleeding disorders occurred in 0.7% of breastfeeding women after the first dose and 0.5% after the second dose. One spontaneous abortion occurred; 93.1% of pregnancies were carried to term, 5.6% late preterm, 0.9% moderate preterm, and 0.3% very preterm. Two children had congenital defects. Vaccine efficacy was 96.3%.

**Conclusion:**

The safety profile of SARS-CoV-2 vaccines in pregnant and breastfeeding women was similar to the general population. Breastfeeding women experienced higher AE rates than pregnant women, presumably due to immune alterations in pregnancy.

## Highlights

• Safety assessment of messenger RNA COVID-19 vaccines in pregnant and breastfeeding women.
• Adverse events are fatigue, headache, myalgia, chills, fever, arthralgia, shivers.
• Less adverse events in pregnant women compared to breastfeeding ones.
• No impact on pregnancy outcomes.

## Introduction

The current SARS-CoV-2 pandemic is not the first in human history, though its impact exceeds other viruses to recently make headlines, whether SARS-CoV-1 [1], Middle East respiratory syndrome [2], or the Hong Kong flu [3]; based on the number of casualties and economic consequence for our globalized world, it is perhaps best compared to the Spanish flu that ravaged the world in the wake of World War I [4]. SARS-CoV-2 has placed an enormous burden on health care systems throughout the world, with hardly any region being unscathed by the pandemic during the past 2 years. Unfortunate impacts on the global economy are evident [5].

SARS-CoV-2 has repeatedly been the reason of lockdowns, contributing to the increased incidence of depression and other mental health problems based on long-term social isolation, while repeated school closings had pronounced consequences for the education of younger generations.

Meanwhile, post-COVID-19 complications and “long COVID” represent a hard-to-quantify challenge for current medicine [6].

Current data mapping the trends of hospitalizations of severe cases and deaths in pregnant women indicate that pregnancy exposes a woman to a higher risk of severe or critical course in case of SARS-CoV-2 infection. For a successful pregnancy, immunological changes are inevitable in order for a woman´s immune system to tolerate the genetically distinct fetus in her womb. These changes in the immune system, as well as alterations in the cardiovascular, respiratory, and other systems can result in increased susceptibility to or increased morbidity and mortality with infection during pregnancy [7]. Similarly, COVID-19 increases the rates of complications and adverse pregnancy outcomes [8]. Although it is impossible to quantify it in detail, even conservative estimates of the worldwide total number of infants with *in utero* exposure to maternal SARS-CoV-2 infection range in the millions [9].

As SARS-CoV-2 has proven to be a persistent issue, vaccination seems to be a promising way out, raising the urgent question of the safety of vaccination for women in vulnerable states, such as pregnancy and/or breastfeeding.

Women are questioning to what extent active immunization is safe under such conditions and what risks it bears for the mother and child. It is essential for primary health care professionals to be able to provide relevant information so that pregnant and breastfeeding women are able to make informed and responsible decisions about whether to get vaccinated or not. In the face of ongoing extensive disinformation campaigns about vaccine safety [10,11], clinicians require adequate data upon which to base their risk-benefit discussions with patients.

The last 25 years of technological advances in molecular biology and biochemistry have accelerated the development of messenger RNA (mRNA) vaccines, resulting in increased interest in the development of mRNA-based therapeutics and prophylactics.

Apart from vaccines against the Zika virus [12,13], Cytomegalovirus [14], or Rabies [15,16] that are being evaluated in clinical trials, the relatively new mRNA-based technology has already been successfully used in oncology [17]. Their immunogenicity and safety profiles in human clinical trials mark mRNA vaccines as a promising avenue of drug development. Together with the potential for generic, low-cost manufacturing processes and the completely synthetic nature, the prospects for mRNA vaccines are promising—if their risk/benefit profiles at least line up with conventional vaccines, or exceed them [18].

Spike protein expression in host cells at the injection site is the desired result mediated through messenger RNA in mRNA vaccines. The spike protein protrudes from the viral surface and is vital for attachment to host cells and penetration [19]. Similarly to other RNA viruses, the SARS-CoV-2 virus has a tendency toward evolution while adapting to the human hosts, resulting in the development of mutations over time. Consequently, several mutant variants of concern with different characteristics than their ancestral strain have emerged [20]. It is often the spike protein that undergoes various mutations, posing a challenge to the prevention and treatment of COVID-19 [21].

Vaccines based on mRNA offer a solution, with the design of new immunogens tailored to emerging strains being time-efficient, as evidenced by the speed with which these vaccines were designed following the inception of the pandemic [22].

The objective of this work was to evaluate the real-world experience of a robust sample of pregnant and breastfeeding women vaccinated against SARS-CoV-2, and to produce a statistically relevant data set useful to clinical practice.

## Methods

### Data collection

An anonymous online questionnaire created via Google Forms was addressed to vaccinated women from Slovakia and Czech Republic, and was used to collect data. Data collection took place from November 19, 2021 until December 31, 2021.

The questionnaire encouraged especially women with preferably two doses of a vaccine, or a single-dose vaccine to respond. It was disseminated through social networks in several groups dedicated to questions of parenthood, mainly in a group for pregnant and lactating women founded by a geneticist invested in online science popularization, further via author's blog and through a gynecologist in Czech Republic. Data about the third dose were not collected, as the registration for the third dose was not yet available in Slovakia at the time of the questionnaire preparation. Therefore, we have queried only the interest to be given the third dose of a vaccine. At the time of the online questionnaire publication on November 19, 2021, the registration for the third dose in Slovakia had just been initiated. Data collection was conducted until the end of December 2021, hence some of the respondents have received the booster (51 respondents [3.2%]).

An English translation of the questionnaire is located in the Supplement (REF).

## Subjects

In total, 2214 pregnant or breastfeeding women who had received at least one dose of a vaccine filled out the questionnaire. Subject disposition is listed in Tables 1 and 2.

**Table 1:** Characteristics of the respondents’ conditions during first and second dose in the sample.

Timing of vaccine doses		Total (n = 2192)	
First dose	Second dose	(%)	(n)
Breastfeeding	Breastfeeding	61.8%	1355
Pregnancy	Pregnancy	27.7%	606
Before pregnancy	Pregnancy	5.0%	110
Pregnancy	*(No second dose)*	3.2%	71
Before pregnancy	Breastfeeding	1.2%	27
Pregnancy	Breastfeeding	1.1%	23

**Table 2:** Comparison of the number of respondents vaccinated with different vaccines, and total number of applied doses.

Vaccine	(%)	Total number of responders/vaccine (n)	Total number of applied doses (n)
Pfizer	85.54%	1839	3614
Moderna	9.67%	207	409
Astra-Zeneca	3.92%	83	166
Janssen (Johnson & Johnson)	0.83%	35	35
Sputnik V	0.05%	1	2

## Analysis

A total of 22 submitted questionnaires with no relevant data entered were excluded. In addition, several non-consistent data were excluded from the analysis too, namely six respondents named their birth years obviously incorrectly (1,2,2020,2021).

Adverse event (AE) incidences were compared using Fisher's exact test. *P*-values are reported without correction for multiplicity; 12 comparisons were made. Statistical analysis was performed using Graphpad Prism v 8.4.3 (Graphpad, CA, USA).

## Outcomes

The primary outcome of this study was to evaluate vaccine safety, specifically mRNA vaccines in the population of pregnant women and breastfeeding women and their breastfed children. The safety was evaluated based on the incidence of AEs and serious AEs (SAEs). The assessed sample consisted of 2192 pregnant and breastfeeding women from Slovakia and Czech Republic.

The secondary outcomes included

1. To assess the trust of respondents toward health care professionals on the basis of the consultation rates prior to the vaccination.
2. To assess whether the vaccination could have any impact on the pregnancy outcomes in pregnant respondents, such as giving birth on the due date/preterm birth date, or the health of infants.
3. To assess vaccine efficacy based on contracting SARS-CoV-2 infection after the vaccination, respectively the course COVID-19.

Assessed baseline variables included

• Age
• Health care background, i.e., education
• SARS-CoV-2 infection prior to vaccination
• Applied vaccines - Pfizer & BioNTech (Comirnaty); Moderna (Spikevax); Astra-Zeneca (Vaxzevria); Johnson & Johnson/Janssen (JCOVDEN); Sputnik Vaccine
• The willingness to be given the booster of the third dose (in case of mRNA vaccines)

The evaluated endpoints were as follows:

Safety in vulnerable patients—primary assessed parameters included

• Safety in pregnant respondents based on AEs incidence
• Safety in breastfeeding respondents based on AEs incidence
• Incidence of SAEs

Pregnant respondents—assessed parameters included

• Date of giving birth + concluded pregnancies vs continuing pregnancies
• Birth of a healthy infant/an ill infant with a confirmed diagnosis of a congenital defect.

## Results

The assessed sample consisted of 2192 respondents of childbearing capacity who filled out the questionnaire after vaccination during pregnancy or breastfeeding.

The mean (SD) age of respondents was 32.6 (4.07) years (range 20-47). 20.3% of respondents had a SARS-CoV-2 infection prior to vaccination. 81% of respondents who were vaccinated at least with one dose during pregnancy consulted their attending gynecologists and/or obstetricians; the majority (66.5%) consulted another healthcare professional prior to vaccination. Of breastfeeding respondents, 36% consulted vaccination with the attending doctor of their child—a pediatrician, respectively a neonatologist. In the last, optional questionnaire question respondents were asked whether they are planning to receive booster vaccination. The question was answered by 73.7% of respondents. The majority of them—73% responded yes; 21.2% responded no; 3.2% that they already had the booster and 2.6% responded that they are shortly after SARS-CoV-2 infection and therefore not planning the third dose now.

Table 1 describes the conditions when the respondents received vaccination (pregnancy, breastfeeding) and their combinations during the first and the second dose.

Table 2 illustrates percentages of respondents vaccinated with individual vaccines. One pregnant respondent received Sputnik V - the first dose in the 36^th^ week and the second dose in the 39^th^ week of pregnancy.

### Safety

**Figure 1:**
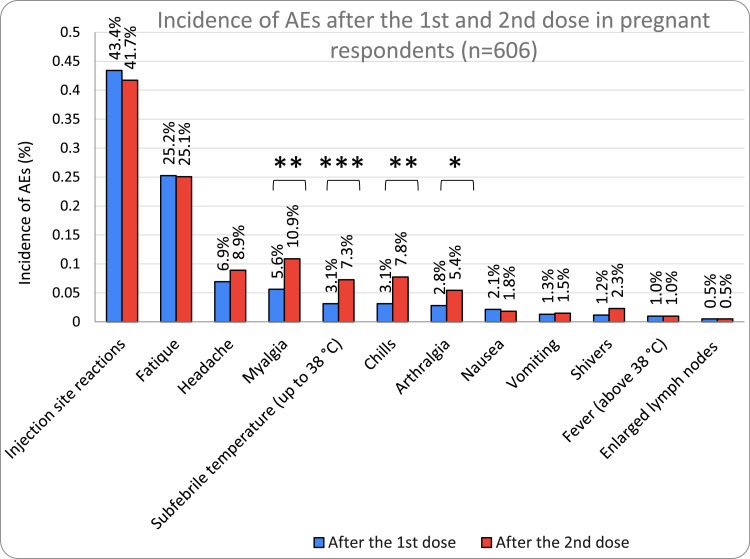
Incidence of AEs after the first and second dose in pregnant respondents (n = 606). AE, adverse event. *: P < 0.05; **: P < 0.01; ***: P < 0.001. Fischer's exact test, two-sided. Not corrected for multiplicity.

We have analyzed the incidence of AEs after the first dose and after the second dose separately; the most frequent AEs are displayed in Figure 1. AEs with incidence below 0.5% are not displayed; these were reported by 1-8 respondents each) (see Supplement for full listing).

All respondents with both doses applied during pregnancy (n = 606) were included in the analysis. After the first dose, 46% of respondents reported that they experienced some AE(s); 47.7% experienced AEs after the second dose. Usually, multiple AEs occurred in parallel. The most frequent AEs were the anticipated injection site reactions (ISR)—sensitivity, pain, swelling, erythema, induration and/or pruritus of the vaccinated arm, followed by fatigue, headache, and myalgia.

**Figure 2:**
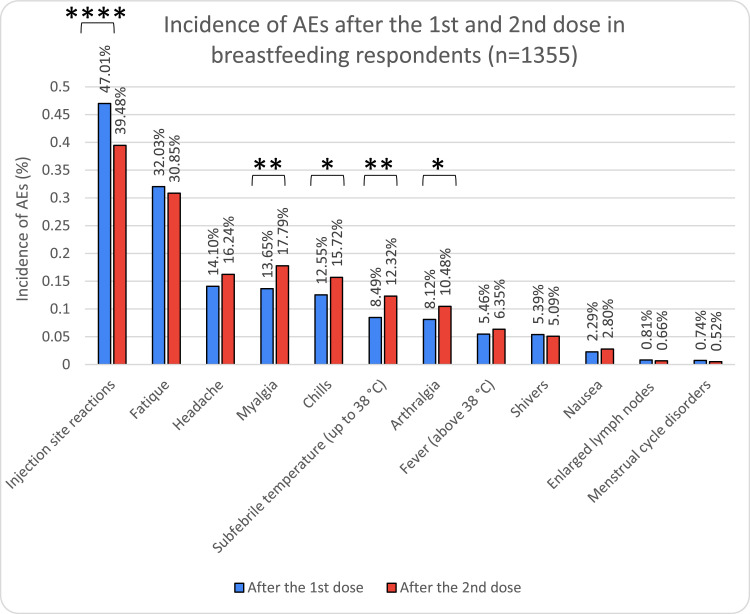
Incidence of AEs after the first and second dose in breastfeeding respondents (n = 1355). AE, adverse event.

Safety analysis of vaccination when both doses were applied, whereas breastfeeding is shown in Figure 2 (n = 1355). AEs were reported by a similarly high number of breastfeeding respondents as in the set of pregnant women—51.4% after the first dose and by 46.2% after the second dose. The most common AEs were similarly the anticipated ISR. Incidence of AEs in breastfeeding women was higher (AEs/respondent = 1.55) than in pregnant women (AEs/respondent = 0.99).

The incidence of ISR was similar in women who were vaccinated, whereas breastfeeding and those who received vaccination while pregnant; other AEs were more common in breastfeeding women. Significant differences in incidence were observed for fatigue (relative risk [RR] 1.230, 95% confidence interval [CI] 1.051-1.444, *P* = 0.0098), headache (RR 1.822, 95% CI 1.379-2.418, *P* <0.0001), myalgia (RR 1.633, 95% CI 1.269-2.110, *P* <0.0001), chills (RR 2.027, 95% CI, *P* <0.0001), body temperature elevation up to 38°C (RR 1.697, 95% CI 1.240-2.335, *P* = 0.0007), arthralgia (RR 1.924, 95% CI 1.340-2.776, *P* = 0.0002), fever >38°C (RR 6.410, 95% CI 2.890-14.30, *P* <0.0001), and shivers (RR 2.204, 95% CI 1.264-3.863, *P* = 0.0049).

The other groups (e.g., participants who received the first dose while pregnant, and the second dose while breastfeeding) did not possess sufficient sample size to allow meaningful statistical comparison of AE incidence.

Breastfeeding women reported menstrual cycle bleeding disorders, such as initiation of the first cycle after the delivery, a skipped cycle, bleeding out of cycle, marked changes in the cycle length (cycle shortening or prolongation), or gynecologic problems with negative microbiology results at a rate of 0.7% after the first dose and 0.5% after the second dose.

The number of respondents who were vaccinated with at least one dose during pregnancy was 810. At the time of answering the questionnaire, 320 of them were already after delivery (39.6%). Table 3 lists the numbers of infants born to these respondents by reached gestational age.

**Table 3:** Infants carried to term/born preterm.

Reached gestational age	Category	n	(%)
≥37 weeks	Term	298	93.1%
34 weeks - <37 weeks	Late preterm	18	5.6%
32 weeks - <34 weeks	Moderate preterm	3	0.9%
31 weeks - <28 weeks	Very preterm	1	0.3%
<28 weeks	Extremely preterm	0	0.0%

Spontaneous abortion in the 26^th^ week of pregnancy was reported by one respondent. She was vaccinated with the first dose before pregnancy and with the second dose in the 9^th^ week of pregnancy. She experienced a severe course of COVID-19 including hospitalization (confirmed by polymerase chain reaction) somewhen after the second vaccine dose. The respondent attributed the abortion to the vaccine, but did not provide any evidence/rationale or statement by health care provider/her attending gynecologist. Another respondent reported a curettage in the 5^th^ week of pregnancy, which could be considered an abortion. She was vaccinated with the first dose before pregnancy and with the second dose in the 5^th^ week of pregnancy. At the time of vaccination, she likely was not aware of the commencing pregnancy or having an early abortion. Two abortions represent 0.2% of all the pregnancies ongoing or completed at the time of answering the questionnaire, where at least one dose was applied during pregnancy (n = 810). Of the respondents who were vaccinated during pregnancy and had already delivered at the time of the questionnaire (n = 320), two reported giving birth to a child with a confirmed congenital defect (0.63%).

The incidence of observed SAEs was 0.3% (7 SAEs in 2165 respondents). In the set of breastfeeding respondents, three SAEs occurred, an acute appendicitis with hospitalization 2 weeks after the first dose; progression of an unspecified autoimmune disorder along with heart palpitation and an unspecified cardiac arrhythmia after the second dose; an unspecified SAE (no other information was provided except that it had taken place). In the set of pregnant respondents, two SAEs occurred, acute nephritis with hospitalization (not otherwise specified); uterine contractions after the second dose applied in the 33^rd^ week of pregnancy but without resulting in preterm birth/abortion—the respondent reported that she gave birth after the completed 37^th^ week of pregnancy and that the baby was healthy without any confirmed diagnosis. In the subset of respondents vaccinated with the first dose before pregnancy and with the second dose during pregnancy, two SAEs occurred as follows: the abovementioned curettage in the 5^th^ week of pregnancy and abortion in the 26^th^ week of pregnancy.

Higher AE incidences were reported for the Spikevax vaccine in comparison to Comirnaty (*P* < 0.0001 for overall AE incidence, ISR, fatigue, headache, myalgia, chills, and elevated temperature; see Supplementary Table 1 for detailed results).

Differences in AE profiles between the first and second dose were observed, mainly driven by the breastfeeding vaccinees are as follows: ISR were more common in this group after the first dose (*P* <0.0001); other AEs were more common after the second dose which include myalgia (*P* = 0.0037), chills (*P* = 0.0205), subfebrile temperatures (*P* = 0.0013), and arthralgia (*P* = 0.0401). Although the incidence of ISR in pregnant vaccinees did not differ between the doses, myalgia (*P* = 0.0011), chills (*P* = 0.0005), subfebrile temperatures (*P* = 0.0017), and arthralgia (*P* = 0.0293) were also more common in the pregnant group after the second dose (see Supplementary Table 2 for detailed results).

### Efficacy

The efficacy of vaccination against SARS-CoV-2 in the sample has shown to be 96.3% (3.7% confirmed SARS-CoV-2 infections after any vaccine dose), corresponding to the efficacy reported by the manufacturers Pfizer-BioNTech a Moderna [23]. As for the severity of the disease, 2.6% experienced an asymptomatic or mild disease course, 1.1% a moderately severe course without hospitalization, and none a severe course that would necessitate hospitalization.

## Discussion

Overall, the safety profile observed in this study sample was similar to that reported in clinical trials of mRNA vaccines against SARS-CoV-2. The AE landscape was dominated by ISR, and classic vaccination reactions, e.g., fatigue, headache, myalgia, chills, and subfebrile temperature elevations or fever. Women who were vaccinated during breastfeeding consistently reported higher rates of AEs than those who were vaccinated during pregnancy; this is likely due to the immune system alterations occurring during pregnancy [24,25]; in particular, this could be attributed to pregnancy-induced changes in cytokine signaling patterns [26], and pregnancy being associated with a propensity toward a T helper 2-type of immune response [27].

No pattern of SAEs was apparent, with most being assessed as chance occurrences consistent with the background incidence. One case of abortion was attributed to vaccination by the respondent, yet no supportive evidence was provided.

Pregnancy, childbirth, and breastfeeding were not negatively impacted by SARS-CoV-2 vaccination. An increase in menstrual cycle disorders was apparent, though - similarly to what other studies report. The Norwegian Institute of Public Health registers increased incidence of various menstrual disturbances among a cohort of 4000 women aged between 18 and 30 years based on the initial results of UngVoksen study, which reported an increase of heavier periods after the first dose, compared to those before vaccination—7.6% before vaccination to 13.6% women after the first dose [28,29], see also, https://www.fhi.no/en/studies/ungvoksen/increased-incidence-of-menstrual-changes-among-young-women/. This ongoing Norwegian study is part of a robust population study monitoring more than 60,000 Norwegian women aged from 18-60 years; the study seeks to clarify whether the cycle disturbances occur more often following vaccination against SARS-CoV-2, as compared to the same women prior to vaccination or to unvaccinated women. Other studies corroborate this evidence [30]. Similarly, the Slovak State Institute for Drug Control has confirmed that amenorrhea and heavy menstrual bleeding after mRNA vaccines have been opened as signals and their causal relationship with vaccination is being reviewed and evaluated (private communication). These data indicate a need for further studies. Although no impact on follicular function was observed following vaccination against SARS-CoV-2 [31], understandable concerns remain because of the distribution of BNT162b2 to ovaries that was observed in preclinical studies [32]. Studies on the impact of vaccination on other ovary parameters, such as ovarian reserve, are ongoing (e.g., NCT04748172).

Transplacental transfer of antibodies can confer protection to neonates while their immune system develops. Although the manifestations of COVID-19 in infants are generally mild, the transferred antibodies could conceivably offer protection against the rare severe manifestations of COVID-19 in infancy [33]; analyses of aggregate data are necessary to assess the magnitude of actual clinical benefit.

Considering that–at least in the present sample–a vast majority (81%) of pregnant women consulted the vaccination with healthcare professionals (which could be interpreted as being in favor of making evidence-based decisions), it is imperative that objective, transparent and detailed data on key aspects of vaccine safety are made available to physicians to facilitate vaccine uptake.

## Conclusion

The present study is a valuable contribution to the safety assessment of SARS-CoV-2 vaccines (including mRNA vaccines) in pregnant and breastfeeding women—a population where structured safety data are difficult to obtain. The dataset is sizable (n = 2192), allowing robust conclusions. The observed AE profile was comparable to that seen in clinical trials in the general population, supporting the notion that SARS-CoV-2 vaccination is reasonably well tolerated by pregnant and breastfeeding women. Pregnant women experienced less AEs than breastfeeding ones, consistent with immune system changes in pregnancy [27]; although antigen uptake may be increased in pregnancy, this is balanced by a suppression of antigen-presenting functions to prevent allogeneic rejection of the fetus [34]. No negative impacts on pregnancy, childbirth and breastfeeding were observed, with the exception of one SAE (spontaneous abortion). An increase in menstrual cycle disorders could be attributed to vaccination, though, warranting further investigation.

## Limitations

The limitation of analyses is the convenience data sample, i.e. that data subject selection was based on the voluntary decision to take part in the survey; no inclusion/ exclusion criteria were applied. Efficacy analysis is limited by several factors, such as the fact that especially pregnant and postpartum respondents intentionally maintained voluntary isolation after vaccination. The study data were recorded before the era of the SARS-CoV-2 omicron variant. The study describes the original monovalent mRNA vaccines; bivalent vaccines were not available at that time. Given the limitation that the questionnaire was filled in by respondents themselves and not by trained personnel, it was not possible to contact the respondents and to resolve queries about data discrepancies, as would be done through electronic case report forms in clinical trials.

## Funding

This research did not receive any specific grant from funding agencies in the public, commercial, or not-for-profit sectors.

## Ethical approval

Data collection and analysis were approved by the Ethics Committee for Biomedical Research of the Faculty of Pharmacy of the Comenius University in Bratislava, Slovakia.

## Author contributions

Terezia Harvanova designed the study, designed and distributed the questionnaire, collected data, and contributed to the manuscript. Petr Novak performed statistical analysis, and compiled the final manuscript. Zuzana Kobliskova contributed to study design and supervised the study.

## Declarations of Competing Interest

Terezia Harvanova does not report any conflict of interest. Petr Novak has received payments from AXON Neuroscience SE and F. Hoffmann-La Roche AG. Zuzana Kobliskova does not report any conflict of interest.

## Appendix Supplementary materials

Image, application 1

Image, application 2
