# Supplementary material for: Evaluation of SARS-CoV-2 vaccination in pregnant and breastfeeding women
Source: IJID Reg. 2023 Jun 14;8:58–63. doi: 10.1016/j.ijregi.2023.06.002 (PMC10265926; doi:10.1016/j.ijregi.2023.06.002)
Supplement: Supplementary file 1 [file mmc1.docx]

Supplement for Harvanova et al., 2023

**Supplementary Figure 1**

Comparisons performed via Fisher’s exact test

** = p < 0.01

*** = p < 0.001

**** = p < 0.0001

**Supplementary Table 1: Comparison of AE incidence between Pfizer & BioNTech (Comirnaty) and Moderna (Spikevax) mRNA SARS-CoV-2 vaccines**

| **Adverse event** | **Comirnaty**  **(%, n = 1839)** | **Spikevax**  **(%, n = 207)** | **Odds ratio** | **95% CI of odds ratio** | **p-value** |
| --- | --- | --- | --- | --- | --- |
| Any adverse event | 55.03 | 79.23 | 0.3208 | 0.1133 to 0.4541 | <0.0001 |
| Injection site reaction | 49.81 | 72.46 | 0.3771 | 0.2743 to 0.5152 | <0.0001 |
| Fatigue | 37.79 | 62.32 | 0.3673 | 0.1365 to 0.4965 | <0.0001 |
| Headache | 17.56 | 32.37 | 0.4452 | 0.3262 to 0.6078 | <0.0001 |
| Myalgia | 17.67 | 39.61 | 0.3272 | 0.2411 to 0.4453 | <0.0001 |
| Chills | 14.25 | 38.16 | 0.2692 | 0.1984 to 0.3664 | <0.0001 |
| Elevated temperature | 11.75 | 29.47 | 0.3185 | 0.2294 to 0.4430 | <0.0001 |

*Fisher’s exact test. Odds ratios < 1 indicate a lower incidence of a given AE with Comirnaty when compared to Spikevax. Pooled analysis of both doses, regardless of pregnancy/nursing status.*

**Supplementary Table 2: Comparison of AE incidences between 1^st^ and 2^nd^ vaccine doses**

|  | Breastfeeding (n = 1355) | | | Pregnant (n = 606) | | | Total (n = 1961 | | |
| --- | --- | --- | --- | --- | --- | --- | --- | --- | --- |
|  | 1^st^ dose | 2^nd^ dose | Odds ratio  (95% CI) | 1^st^ dose | 2^nd^ dose | Odds ratio  (95% CI) | 1^st^ dose | 2^nd^ dose | Odds ratio  (95% CI) |
| Any adverse event | 697 | 626 | 1.234  (1.061 to 1.435)  p = 0.0071 | 279 | 289 | 0.9359  (0.7468 to 1.172)  p = 0.6044 | 976 | 915 | 1.133  (1.000 to 1.283)  p = 0.0552 |
| Injection site reactions | 637 | 535 | 1.360  (1.166 to 1.583)  **p < 0.0001** | 263 | 253 | 1.070  (0.8504 to 1.346)  p = 0.6011 | 900 | 788 | 1.263  (1.1125 to 1.4332)  **p = 0.0003** |
| Fatigue | 434 | 418 | 1.056  (0.8993 to 1.241)  p = 0.5349 | 153 | 152 | 1.009  (0.7752 to 1.313)  p > 0.9999 | 587 | 570 | 1.043  (0.9088 to 1.196)  p = 0.5753 |
| Headache | 191 | 220 | 0.8466  (0.6869 to 1.047)  p = 0.1337 | 42 | 54 | 0.7612  (0.4955 to 1.146)  p = 0.2419 | 233 | 274 | 0.8302  (0.6877 to 1.000)  p = 0.0569 |
| Myalgia | 185 | 241 | 0.7309  (0.5948 to 0.8995)  p = 0.0037 | 34 | 66 | 0.4863  (0.3123 to 0.7517)  **p = 0.0011** | 219 | 307 | 0.6773  (0.5617 to 0.8167)  **p < 0.0001** |
| Chills | 170 | 213 | 0.7692  (0.6186 to 0.9583)  p = 0.0205 | 19 | 47 | 0.3850  (0.2278 to 0.6551)  **p = 0.0005** | 189 | 260 | 0.6978  (0.5722 to 0.8511)  **p = 0.0004** |
| Higher body temperature (up to 38 °C) | 115 | 167 | 0.6597  (0.5134 to 0.8466)  **p = 0.0013** | 19 | 44 | 0.4196  (0.2434 to 0.7233)  **p = 0.0017** | 134 | 211 | 0.6083  (0.4855 to 0.7647)  **p < 0.0001** |
| Arthralgia | 110 | 142 | 0.7547  (0.5805 to 0.9826)  p = 0.0401 | 17 | 33 | 0.5012  (0.2835 to 0.9195)  p = 0.0293 | 127 | 175 | 0.7067  (0.5558 to 0.8978)  p = 0.0048 |
| Fever (above 38 °C) | 74 | 86 | 0.8524  (0.6210 to 1.173)  p = 0.3700 | 6 | 6 | 1.000  (0.3172 to 3.152)  p > 0.9999 | 80 | 92 | 0.8640  (0.6329 to 1.172)  p = 0.3911 |
| Shivers | 73 | 69 | 1.061  (0.7524 to 1.479)  p = 0.7960 | 7 | 14 | 0.4942  (0.1846 to 1.218)  p = 0.1854 | 80 | 83 | 0.9623  (0.7053 to 1.311)  0.8729 |

Odds ratios < 1 indicate the given AE is more frequent after the 2^nd^ dose; odds ratios > 1 indicate the given AE is more frequent after the 1^st^ dose. P-values are reported without correction for multiplicity; 30 comparisons were made. P-values that are significant at p < 0.05 even after Bonferroni correction are highlighted in **bold.**
