# Supplementary material for: Evaluation of SARS-CoV-2 vaccination in pregnant and breastfeeding women
Source: IJID Reg. 2023 Jun 14;8:58–63. doi: 10.1016/j.ijregi.2023.06.002 (PMC10265926; doi:10.1016/j.ijregi.2023.06.002)
Supplement: Supplementary file 2 [file mmc2.docx]

Supplement for “Evaluation of vaccination against SARS-CoV-2 in pregnant and breastfeeding women”

2022-09-23

# Questionnaire

*The questionnaire consisted of 84 questions, with sections being assembled into interactive logical sequences of questions, so that each respondent filled in only the questions referring to her specific case. It branched out based on the time when the woman was given the first dose or the second vaccine dose (before pregnancy/during pregnancy/after the giving birth). The introduction consisted of 4 general questions:*

1. *For age calculation, women were asked to* enter the year and month of birth. *To preserve anonymity the birthday was not collected.*

2. Were you diagnosed with COVID-19 or were you positively tested for SARS-CoV-2 prior to vaccination? (select yes only if it was confirmed by PCR/antigen test). (yes/no)

3. Do you have a healthcare background/natural sciences field or do you study it? (such as nurse, pharmacist, doctor, dentist, midwife et cetera). (yes/no)

4. Have you consulted any doctor or health care professional prior to vaccination? (yes/no)

If yes, who was it? (if “other”, please describe).

- Gynaecologist
- General physician
- Paediatrician
- Pharmacist
- other – describe *(text field)*

Your state during the first dose - when did you please take the first dose? (or a single dose vaccine?)

- Before pregnancy
- During pregnancy
- Post partum

**The section for mothers vaccinated during pregnancy consisted of the following questions:**

Which vaccine did you receive?

- Pfizer-BioNTech
- Moderna
- Astra Zeneca
- Janssen (Johnson & Johnson)
- Sputnik V

In case of Pfizer-BioNTech, Moderna, Astra Zeneca and Sputnik V – have you already received both doses? (yes/no)

In which week did you take the first dose/single-dose vaccine?

*(drop down menu with weeks of pregnancy)*

Have you experienced any adverse reactions after the first dose/single-dose vaccine? (yes/no)

If yes, what was it?

- Arm Pain/swelling/sensitivity
- Fever (up to 38 °C)
- Fever (above 38 °C)
- Fatigue
- Chills
- Muscle pain
- Headache
- Joint pain
- Nausea
- Vomiting
- Shivers
- other - please fill in… *(text field)*

If yes, did you report the adverse reaction to your attending physician or eventually directly to ŠUKL (Slovak regulatory authority)? (yes/no)

Have you experienced any SERIOUS adverse reactions after the first dose/single-dose vaccine (e.g., one that resulted in hospitalisation/prolonged hospitalisation/was life threatening or has led to persistent injury in you and/or your child? (yes/no)

- If yes, please fill in what it was: (text field)
- If yes, did you report this adverse reaction to your attending physician or eventually directly to ŠUKL (Slovak regulatory authority)? (yes/no)

Have you already received the second dose, too? (yes/no) *+ the same questions related to adverse reaction incidence.*

Are you already post partum? (yes/no)

If yes, when did you give birth?

- after the 38th pregnancy week *(this would open the section for children of fully vaccinated mothers)*
- before the finished 38th pregnancy week *(this would open the section for pre-term children)*

*Birth before due date*:

If the child was born pre-term, in which week of pregnancy was it born? *(drop-down menu with weeks of pregnancy)*

Did you give birth to a healthy child?

- Yes
- No, the child has a confirmed diagnosis/diagnoses
  - If a diagnosis was confirmed, please describe: *(text field)*

**The section for mothers vaccinated after childbirth contained the following questions:**

Which vaccine did you receive?

- Pfizer-BioNTech
- Moderna
- Astra Zeneca
- Janssen (Johnson & Johnson)
- Sputnik V

In case of Pfizer-BioNTech, Moderna, Astra Zeneca and Sputnik V – have you already received both doses? (yes/no)

Have you been breastfeeding at the time when you received the FIRST dose of the vaccine? (yes/no)

Did you experience any adverse effects after the FIRST vaccine dose (or the only dose)? (yes/no)

- Arm Pain/swelling/sensitivity
- Fever (up to 38 °C)
- Fever (above 38 °C)
- Fatigue
- Chills
- Muscle pain
- Headache
- Joint pain
- Nausea
- Vomiting
- Shivers
- other - please fill in… *(text field)*

If yes, did you report the adverse reaction to your attending physician (GP, gynaecologist, paediatrician) or eventually directly to ŠUKL (Slovak regulatory authority)? (yes/no)

Have you experienced any SERIOUS adverse reactions after the FIRST dose/single-dose vaccine (e.g., one that resulted in hospitalisation/prolonged hospitalisation/was life threatening or has led to persistent injury in you and/or your child? (yes/no)

- If yes, please fill in what it was: *(text field)*
- If yes, did you report this adverse reaction to your attending physician or eventually directly to ŠUKL (Slovak regulatory authority)? (yes/no)

*For those who received the second dose after giving birth, the questions were similar, with identical options.*

**At the end of the questionnaire, each respondent who received at least one vaccine dose was asked about whether she had COVID after vaccination:**

Were you diagnosed with COVID-19 / were you tested positive for SARS-CoV-2 after vaccination? Choose “yes” only if the infection was confirmed by a test.

- No
- Yes – after the first dose
- Yes – after the second dose

What test was used to confirm COVID-19?

- PCR test
- antigen test at a testing point
- home antigen test

If you chose “yes” above, what was the disease course?

- No symptoms / mild
- More serious, but no hospitalisation was needed
- Serious disease course – I had to be hospitalised

*The last question pertained to the willingness to receive a third dose:*

Do you plan on receiving a third dose, or have you already received it?

- Yes, I plan to
- No, I don’t
- I have received the third dose already
- I have recent had COVID-19, thus I don’t plan on receiving the third dose now.

*The questionnaire furthermore contained thanks for the time investment and the respondent’s contribution to statistical analysis of vaccine safety and efficacy, and the possibility to aid women who are undecided yet.*

*Respondents were informed about the purpose of the questionnaire, and that data would be processed in line with the currently valid laws of the Slovak Republic and European Union, including GDPR.*

# AEs by SOC (full listing)

| **System Organ Class  PT** | **pregnant (N=606)** | | **lactation (N=1355)** | | **other (N=231)** | | **total (N=2192)** | |
| --- | --- | --- | --- | --- | --- | --- | --- | --- |
|  | **n** | **%** | **n** | **%** | **n** | **%** | **n** | **%** |
| **Infections and infestations** |  |  |  |  |  |  |  |  |
| Mastitis |  |  | 6 | 0.44% |  |  | 6 | 0.27% |
| Nasopharyngitis | 1 | 0.17% | 3 | 0.22% |  |  | 4 | 0.18% |
| Oral herpes |  |  | 3 | 0.22% |  |  | 3 | 0.14% |
| Ear infection |  |  | 2 | 0.15% |  |  | 2 | 0.09% |
| Appendicitis |  |  | 1 | 0.07% |  |  | 1 | 0.05% |
| Sinusitis |  |  |  |  | 1 | 0.43% | 1 | 0.05% |
| Tetanus |  |  | 1 | 0.07% |  |  | 1 | 0.05% |
| **Neoplasms benign, malignant and unspecified (incl. cysts and polyps)** |  |  |  |  |  |  |  |  |
| Infected nevus |  |  | 1 | 0.07% |  |  | 1 | 0.05% |
| **Blood and lymphatic system disorders** |  |  |  |  |  |  |  |  |
| Lymphadenopathy | 6 | 0.99% | 20 | 1.48% | 1 | 0.43% | 27 | 1.23% |
| **Immune system disorders** |  |  |  |  |  |  |  |  |
| Autoimmune disorder |  |  | 1 | 0.07% |  |  | 1 | 0.05% |
| **Psychiatric disorders** |  |  |  |  |  |  |  |  |
| Insomnia | 2 | 0.33% | 3 | 0.22% |  |  | 5 | 0.23% |
| Anxiety | 2 | 0.33% | 2 | 0.15% |  |  | 4 | 0.18% |
| Anger |  |  | 2 | 0.15% |  |  | 2 | 0.09% |
| **Nervous system disorders** |  |  |  |  |  |  |  |  |
| Headache | 96 | 15.84% | 411 | 30.33% | 25 | 10.82% | 532 | 24.27% |
| Paraesthesia |  |  | 7 | 0.52% | 1 | 0.43% | 8 | 0.36% |
| Dizziness | 1 | 0.17% | 6 | 0.44% |  |  | 7 | 0.32% |
| Migraine | 2 | 0.33% |  |  | 1 | 0.43% | 3 | 0.14% |
| Monoplegia |  |  | 1 | 0.07% |  |  | 1 | 0.05% |
| Seizure |  |  | 1 | 0.07% |  |  | 1 | 0.05% |
| Transient aphasia | 1 | 0.17% |  |  |  |  | 1 | 0.05% |
| **Eye disorders** |  |  |  |  |  |  |  |  |
| Eye irritation | 2 | 0.33% |  |  |  |  | 2 | 0.09% |
| Eye pain |  |  | 1 | 0.07% |  |  | 1 | 0.05% |
| Vision blurred | 1 | 0.17% |  |  |  |  | 1 | 0.05% |
| **Cardiac disorders** |  |  |  |  |  |  |  |  |
| Tachycardia | 6 | 0.99% | 9 | 0.66% | 1 | 0.43% | 16 | 0.73% |
| Arrhythmia | 1 | 0.17% | 2 | 0.15% |  |  | 3 | 0.14% |
| Bradycardia | 1 | 0.17% |  |  |  |  | 1 | 0.05% |
| **Vascular disorders** |  |  |  |  |  |  |  |  |
| Flash hot |  |  | 2 | 0.15% | 1 | 0.43% | 3 | 0.14% |
| Haematoma |  |  | 2 | 0.15% |  |  | 2 | 0.09% |
| **Respiratory, thoracic and mediastinal disorders** |  |  |  |  |  |  |  |  |
| Dyspnoea | 1 | 0.17% | 2 | 0.15% |  |  | 3 | 0.14% |
| Cough | 1 | 0.17% | 1 | 0.07% |  |  | 2 | 0.09% |
| Oropharyngeal pain |  |  | 1 | 0.07% |  |  | 1 | 0.05% |
| **Gastrointestinal disorders** |  |  |  |  |  |  |  |  |
| Nausea | 24 | 3.96% | 69 | 5.09% | 7 | 3.03% | 100 | 4.56% |
| Vomiting | 17 | 2.81% | 16 | 1.18% | 1 | 0.43% | 34 | 1.55% |
| Diarrhoea | 4 | 0.66% | 5 | 0.37% | 1 | 0.43% | 10 | 0.46% |
| Dyspepsia | 1 | 0.17% | 4 | 0.30% |  |  | 5 | 0.23% |
| Abdominal pain |  |  | 1 | 0.07% |  |  | 1 | 0.05% |
| Regurgitation |  |  | 1 | 0.07% |  |  | 1 | 0.05% |
| **Skin and subcutaneous tissue disorders** |  |  |  |  |  |  |  |  |
| Hyperhidrosis | 2 | 0.33% | 4 | 0.30% |  |  | 6 | 0.27% |
| Rash/Eczema |  |  | 4 | 0.30% |  |  | 4 | 0.18% |
| **Musculoskeletal and connective tissue disorders** |  |  |  |  |  |  |  |  |
| Myalgia | 100 | 16.50% | 426 | 31.44% | 22 | 9.52% | 548 | 25.00% |
| Arthralgia | 50 | 8.25% | 252 | 18.60% | 15 | 6.49% | 317 | 14.46% |
| Back pain | 3 | 0.50% | 3 | 0.22% | 2 | 0.87% | 8 | 0.36% |
| Musculoskeletal stiffness |  |  | 1 | 0.07% |  |  | 1 | 0.05% |
| **Renal and urinary disorders** |  |  |  |  |  |  |  |  |
| Cystitis noninfective | 1 | 0.17% |  |  |  |  | 1 | 0.05% |
| Nephritis | 1 | 0.17% |  |  |  |  | 1 | 0.05% |
| **Pregnancy, puerperium and perinatal conditions** |  |  |  |  |  |  |  |  |
| Abortion early |  |  |  |  | 1 | 0.43% | 1 | 0.05% |
| Uterine contractions during pregnancy | 1 | 0.17% |  |  |  |  | 1 | 0.05% |
| **Reproductive system and breast disorders** |  |  |  |  |  |  |  |  |
| Menstrual disorder |  |  | 17 | 1.25% |  |  | 17 | 0.78% |
| Suppressed lactation |  |  | 7 | 0.52% |  |  | 7 | 0.32% |
| Breast pain |  |  | 2 | 0.15% |  |  | 2 | 0.09% |
| Galactorrhoea |  |  | 1 | 0.07% |  |  | 1 | 0.05% |
| Galactostasis |  |  | 1 | 0.07% |  |  | 1 | 0.05% |
| **General disorders and administration site conditions** |  |  |  |  |  |  |  |  |
| Vaccination site reaction* | 516 | 85.15% | 1172 | 86.49% | 79 | 34.20% | 1767 | 80.61% |
| Asthenia | 152 | 25.08% | 852 | 62.88% |  |  | 1004 | 45.80% |
| Chills/ Feeling cold | 66 | 10.89% | 383 | 28.27% | 18 | 7.79% | 467 | 21.30% |
| Fatigue | 153 | 25.25% |  |  | 56 | 24.24% | 209 | 9.53% |
| Pyrexia | 12 | 1.98% | 160 | 11.81% | 5 | 2.16% | 177 | 8.07% |
| Chills | 21 | 3.47% | 142 | 10.48% | 7 | 3.03% | 170 | 7.76% |
| General discomfort | 1 | 0.17% | 6 | 0.44% |  |  | 7 | 0.32% |
| Chest discomfort | 2 | 0.33% | 4 | 0.30% |  |  | 6 | 0.27% |
| Non-cardiac chest pain |  |  | 2 | 0.15% |  |  | 2 | 0.09% |
| Adverse event |  |  | 1 | 0.07% |  |  | 1 | 0.05% |
| Peripheral swelling |  |  | 1 | 0.07% |  |  | 1 | 0.05% |
| **Investigations** |  |  |  |  |  |  |  |  |
| Body temperature increased | 63 | 10.40% | 282 | 20.81% | 17 | 7.36% | 362 | 16.51% |
| **Surgical and medical procedures** |  |  |  |  |  |  |  |  |
| Uterine dilation and curettage |  |  |  |  | 1 | 0.43% | 1 | 0.05% |
| * this includes PTs: Injection site pain, Injection site oedema and Injection site irritation "other" includes all respondents who did not receive both doses during pregnancy or breastfeeding - e.g., those who had one dose during pregnancy, and the other after giving birth | | | | | | | | |
